# Case of Tetanus

**Published:** 1810-07

**Authors:** R. Jones

**Affiliations:** Surgeon


### *Case of Tetanus.* By Mr. R. Jones, Surgeon.

MRS. MARTIN, residing at No. 4, Bowl and Pin Alley, aged about 52 years, in going through Old Boswell Court, on Monday the 23d of April, was wounded in the left cheek by an arrow, shot by a little boy; which was formed of a stick sharply pointed. The point of it, about half an inch in length, remained fixed in the cheek, and had penetrated the masseter muscle, but not deeply, being rather in a slanting direction. She called upon me about ten minutes after the accident. I extracted the portion fixed, and a little blood issued out. I dressed it simply, and desired her to call upon me the next morning. She however returned the evening of the same day a little agitated; described herself as feeling much pain in the wound, and a dimness in the sight of the left eye, of which, she feared she should lose the use. I told her to poultice the wound, and to call upon me in the morning; and solaced her with the hopes of no such disagreeable consequence happening. I heard no more of her till Wednesday evening about ten o'clock, when she sent for me. I found her suffering under very violent attacks of tetanus, and a great degree of trismus. The flexors of the lower extremities were quite rigid; abdomen tense; and whenever the spasms came on, which was every five or ten minutes, the actions of the diaphragm and abdominal muscles seemed particularly to distress her. She often laid hold of my hand and applied it to her abdomen, and a loud gurgling noise was heard, as if from the undulation of water. The muscles of the neck and face were rigid, and sometimes contorted, particularly on the left side, and an efflorescence suddenly appeared and disappeared. As she had no evacuation since Monday, I ordered her a glyster; also a mixture, containing ninety drops of tinct. opii, to take a fourth part every two hours, and opening pills, composed of calomel, gr. x. Ext. coloc. c. ʒi. Opii purif. gr. iij. ft. pil xij. Cap. iij. 6ta. quaque hora.

Thursday 26. I was this day informed by her attendants, rather imperfectly, of the progress of this disease from Monday to Wednesday evening. It appears she was attacked a few hours after I saw her on Monday night, with what they called cramps, &c. She immediately sent for me, but from some cause or other, the messenger went elsewhere. When I called this morning, I found her suffering under an aggravation of all the symptoms; sensible to to every thing that was said to her, but speechless; she took a part of her medicine with difficulty, but could not be persuaded to have the glyster. I endeavoured to get down some medicine; and after much difficulty, she swallowed a pill of five grains of calomel, and about eighty or ninety drops of tinct. opii. I also ordered her neck, chest, and abdomen to be fomented. She retained the greatest part of a glyster, that was administered this day. When I saw her again in the evening, every symptom was aggravated; and every attempt at deglutition was as distressing as it was unavailing; all I could hope for, was to get down the five grain pills of calomel, to each of which I now united one grain of antim. tart. with directions to take them every six hours, and to wash them down with about a drachm of tinct opii. diluted with each dose. She had had no stool to day, but passed her urine freely.

Friday 27. The patient had in the course of last night and this morning vomited up some black mucous substance, and passed a stool of the same appearance. The tetanic symptoms, though very violent in the night, are certainly not so now. We were able to get down the medicines with tolerable ease. In the evening she wrote down, that she should like some collared eels, and a little porter. They were introduced in the same manner that her medicine is administered, through the vacancy left by the loss of her upper front teeth. She was able to take a little, and it was the only nourishment she had taken for three days. Her jaws were completely locked these two days, except when they were forced open by the spasms, yet she appeared to all about her better this evening.

Saturday 28. When I visited her this morning she was in a most profuse perspiration, with the affected muscles as rigid and contracted as ever. Her lower extremities were drawn up towards her body. She attempted to take some tea but could not; though at particular times, I was told, she swallowed with tolerable ease; but whenever I saw her attempt it, the spasms of the fauces and throat were very distressing, and much resembled that of a person suffering from hydrophobia. When I saw her again this evening, she perspired freely, and made signs that she was better. Her medicines are continued, but with less calomel, and the antim. tart. is entirely omitted.

Sunday 29. She is this morning better; has had no severe spasms since Friday night. She articulates more distinctly. Her attendants are able to introduce the point of a tea spoon through the vacancy left by the loss of her upper front front teeth, and in that manner she has sucked about two-thirds of the yolke of an egg, and some porter; but the most promising announcement of this day is, the absence of those distressing spasms that accompanied every effort at deglutition. Her sight is also much recovered, but as the calomel did not act upon the bowels, I ordered this morning a cathartic mixture. About two o'clock in the afternoon, she experienced a most violent attack of spasms, commencing in the wounded cheek, and instantaneously attacking the left side of the neck and arm, down to the fingers. In the course of a minute or two they became inflamed, and in about fifteen minutes after were completely black. This blackness seemed to proceed in the course of the nerves of the left brachial plexus, the first or axillary nerve of which had been so affected the day before; but the whole body seemed writhed with the spasms. She became tranquil again in the evening.

Monday 30. The blackness in the arm is gone off, but around the left eye and cheek a similar affection took place; which terminated in the same manner. She has had three copious evacuations during the night; has taken nourishment, and feels considerably better. She can now distinctly describe her wants and feelings, as her tongue seems now completely released. We could not prevail upon her to take her medicines regularly these two days, as she has discovered that laudanum is given to her; to which she has a particular aversion.

Tuesday, May 1st. Continues better; has had more evacuations, and also vomited once. For these two days she has complained of a pain extending from the wound, which is completely healed, to the mouth, and of a disagreeable smell. Her jaws remained closed, although she has attempted to separate them by a variety of instruments.

Wednesday 2. She was by no means so well this morning; had been much troubled with spasms last night, which partially attacked her throat. The pain in the wounded cheek was, at times, very acute; and like nothing, as she said, that she could describe. She felt pain also in the other side, proceeding along the upper lip, and over the arch of the nose; but by no means of equal violence. She vomited once, and had several motions. I thought proper to persist in the exhibition of mercury; but from the state of her stomach and bowels, preferred the friction. A drachm of ung. hydr. fort. with ten grains of camphor, was directed to be rubbed inside of the thigh every night and morning. The left hand was last night so clenched, that that her nails had penetrated the skin; but I could move the first and second joints of the fingers this morning.

Thursday 3. She was, upon the whole, better to day, but felt much pain at the soles of her feet.

Friday 4. Still better. The ointment is readily absorbed, and she has had no severe spasms since Wednesday night. The trismus continues, and her extremities much in the same state. She has taken some rice milk and porter, to which she is very partial; and though she swallows with a great deal more ease, yet there is still a difficulty.

Saturday 5. Much the same as yesterday. The quantity of calomel she had before taken, with the subsequent inunction of the ointment, has brought on a ptyalism. Her gums do not appear much affected, but as she feels a soreness about her mouth, and her tongue is swelled, I have desisted from the friction. The only amelioration that I could mark to day, is a greater freedom in the knee joints.

Sunday 6. Better in respect to the tetanic symptoms, but had been several times sick, and rejected the slight nourishment she had taken within the last twenty-four hours. Complains more than usual of the pain in the left hypochondrium; and whenever she closes her left eye, the most horrid objects present themselves to her. A large blister applied to the nape of the neck.

Monday 7. She was much harrassed all this day with purging and vomiting, and appears completely under the influence of mercury.

Tuesday 8. Much better this morning. The rigidity of the flexors of the lower extremities is sensibly less. She complains of considerable pain about the umbilicus, and has now quite a sore mouth. There is still much irritability of the stomach.

Thursday 10. The sickness now and then recurs, with some irritation of the bowels; but the acute pain which she felt at the umbilicus is going off. The whole of the body is this day covered with an efflorescence, and the mouth exhibits the mercury in full force. She has now the perfect use of both her arms. It may not be amiss to remark, that the eyes, during the whole of this attack, never twinkled, and the eye-lids could only be brought together by the fingers.

Saturday 12. My patient had been a great deal better the last three days, was even able to sit up in bed, and talked of getting up to have her bed made; but last night she was exposed to a current of air through a broken pane pane of glass; it fell, she says, upon the wounded cheek, and afterwards through her whole body. She is not so well this morning.

Sunday 13. Passed an indifferent night, and is visibly affected with cold. She has a cough upon her, and considerable pain at her left side. Her pulse is not much affected, nor has it brought on any spasms, though I rather fear them. There is a slight tremulation in all the muscles of her body. She cannot retain any thing on her stomach, nor can I get her to take opium in any quantity.

Monday 14. The spasms came on last night, and continued the whole of this day in the most violent degree, particularly in the abdominal muscles and diaphragm. In the course of the evening she threw up some black jelly-like looking substance, and was relieved. I ordered the abdomen to be again fomented, and allowed her as much Hollands or wine as her stomach would bear. She continues to have a free discharge of saliva, and her bowels are kept moderately open.

Tuesday 15. She is this morning considerably better; the spasms do not recur so often, nor continue violent.

Saturday 19 Continues amending, but the least exposure to cold is apt to bring on some degree of spasm. She is however infinitely better than she was on the Monday.

Thursday 24. My patient is now so much amended that I have discontinued my regular visits. I do not consider her recovery as at all doubtful; but am perfectly aware that she will for some time to come, feel the effects of so violent an attack. She has little or no discharge of saliva, and can now separate her jaws to some extent.

Having traced this strongly marked and generally fatal complaint to a favourable issue, a variety of remarks and suggestions will naturally arise as to the success of the method of treatment adopted in this Case.

Sir William Blizard, in his Lecture on Tetanus, at the College, has favoured us with one or two cases successfully treated by very strong doses of calomel and opium, and an inordinate allowance of wine; I believe, to one patient, three bottles a day, for some days. I should infer the fatal termination of this complaint in general, to be owing, in a great measure, to a total cessation or derangement of the functions of the viscera; for when, after the tartar emetic, and subsequently from other causes, my patient vomited that black offensive matter, she was visibly better. I do not recollect the tartarised antimony being recommended by any author, but I am disposed to think think very favourably of it in the first stage of the complaint. Opium has been long in much repute; circumstances did not admit its being given to that extent as to warrant me to attribute any thing to it in this instance. Not so with my principal remedy, for I am disposed to speak very highly of hydrargyrus. As soon as the patient was under its influence, the spasms gradually gave way; but it would be needless to add, after what I have before stated, of what serious importance it is, that the patient should not be exposed to cold. I cannot, for this reason, at all recommend the cold bath. In one instance to my knowledge, at a public hospital, a few months ago, a patient was put into a cold bath, and she died a few hours after; but this should not deter us from trying it in idiopathic tetanus. Before I conclude, I must again exhort most particular attention to be paid to the stomach and bowels in tetanus; an advice particularly recommended by Sir Wm. Blizard; and one, which I repeatedly witnessed the good of attending to throughout this case.

*Chancery Lane, May* 29, 1810.

